# Caspase-5: Structure, Pro-Inflammatory Activity and Evolution

**DOI:** 10.3390/biom14050520

**Published:** 2024-04-26

**Authors:** Leopold Eckhart, Heinz Fischer

**Affiliations:** 1 Department of Dermatology, Medical University of Vienna, 1090 Vienna, Austria; 2 Division of Cell and Developmental Biology, Center for Anatomy and Cell Biology, Medical University of Vienna, 1090 Vienna, Austria; heinz.fischer@meduniwien.ac.at

**Keywords:** caspase, inflammasome, inflammation, gasdermin, evolution, gene duplication, endotoxin, lipopolysaccharide, sepsis, pyroptosis

## Abstract

Caspase-5 is a protease that induces inflammation in response to lipopolysaccharide (LPS), a component of the cell envelope of Gram-negative bacteria. The expression level of the *CASP5* gene is very low in the basal state, but strongly increases in the presence of LPS. Intracellular LPS binds to the caspase activation and recruitment domain (CARD) of caspase-5, leading to the formation of a non-canonical inflammasome. Subsequently, the catalytic domain of caspase-5 cleaves gasdermin D and thereby facilitates the formation of cell membrane pores through which pro-inflammatory cytokines of the interleukin-1 family are released. Caspase-4 is also able to form a non-canonical inflammasome upon binding to LPS, but its expression is less dependent on LPS than the expression of caspase-5. Caspase-4 and caspase-5 have evolved via the duplication of a single ancestral gene in a subclade of primates, including humans. Notably, the main biomedical model species, the mouse, has only one ortholog, namely caspase-11. Here, we review the structural features and the mechanisms of regulation that are important for the pro-inflammatory roles of caspase-5. We summarize the interspecies differences and the evolution of pro-inflammatory caspases in mammals and discuss the potential roles of caspase-5 in the defense against Gram-negative bacteria and in sepsis.

## 1. Introduction: Caspases Are Critical Regulators of Apoptosis and Pyroptosis

Caspases are cysteine proteases that cleave their substrates after aspartic acid residues [1,2,3,4]. Humans have eleven caspases that are catalytically active, namely caspase-1 through 10 and caspase-14. The phylogenetic analysis of caspase genes has defined three clades of caspases: (I) caspase-1, -2, -4, -5, -9 and -14, (II) caspase-3, -6 and -7 and (III) caspase-8 and -10 [5]. The catalytic activity of caspases resides in the caspase domain (pfam00656: peptidase_C14) [6], which is well-conserved, whereas the protein segment on the amino-terminal side of this domain differs among caspases. All caspases of clade I except caspase-14 [7] contain a caspase recruitment domain (CARD), caspases of clade III contain two death effector domains (DED) and other caspases have a short amino-terminal region that does not fold into a conserved domain. Functionally, capase-2, -3, -6, -7, -8, -9 and -10 are implicated in the induction of apoptosis, a mode of programmed cell death [8]; caspase-14 is involved in the terminal differentiation of epidermal keratinocytes towards the cornified layer on the skin surface [9,10]; and caspase-1, -4 and -5 are primarily activators of pro-inflammatory signaling, but also induce a form of programmed cell death, named pyroptosis [8].

The proteolytic activity of caspases depends on a cysteine at the active site. Together with a histidine, it forms the catalytic dyad [3]. An arginine in the carboxy-terminal region of caspases is essential for substrate binding [1,3,4]. These three residues are conserved in all active caspases but not in the caspase-8-like protein c-FLIP, which functions as an inhibitor of caspase-8 [3,11]. Individual caspases differ in their substrate specificity, which is largely determined by the presence and accessibility of a 4-amino residue motifs ending with aspartic acid [12,13,14]. Caspase-3 and caspase-7 cleave a broad spectrum of proteins and these cleavages typically lead to the demise of the cell, known as apoptosis. Other caspases, such as caspase-14 [10], have a narrower range of substrate proteins. Overexpression studies in cultured cells can predict many substrates, provided that these proteins are constitutively expressed in the cells [12].

In addition to the substrate specificity, the mechanism of activation determines the functions of individual caspases. The prototypical caspases with a CARD (caspase-1, caspase-9) or two DED folds (caspase-8) in their prodomain bind other proteins through homotypic CARD–CARD or DED–DED interactions to form large protein complexes known as inflammasomes (caspase-1), PIDDosome (caspase-2), apoptosome (caspase-9), or the death-inducing signaling complex (caspase-8) [15,16,17]. The formation of these complexes depends on specific stimuli and subsequently triggers activation of the caspase within the complex. Thus, complex formation is a major regulatory step that integrates the function of caspases into cellular processes. Caspase-3, -6 and -7 lack a long prodomain and are activated by proteolytic cleavage through other caspases, thereby amplifying the total proteolytic activity [18,19]. Caspase-14 is activated by dimerization under the unique (kosmotropic) conditions of terminally differentiated keratinocytes in which it is expressed [20,21]. 

Caspases are critical regulators of important cellular processes that are linked to the development and turnover of tissues as well as defense against infections. Capase-2, -3, -6, -7, -8, -9 and -10 are the characteristic mediators of the prototypical form of programmed cell death, that is, apoptosis. In response to extracellular (e.g., Fas ligand) or intracellular (e.g., the release of cytochrome c into the cytoplasm) cues, caspases cleave structural and regulatory proteins, leading to the death of the cell, which is subsequently phagocytosed by macrophages or other cells. Notably, caspase-8 plays a dual role by inducing apoptosis and, via cleavage of RIPK1, suppressing pro-inflammatory necroptosis [22,23]. The activation of caspase-1, -4 and -5 induces pyroptosis [2]. This pro-inflammatory mode of cell death involves the proteolytic processing of gasdermins, enabling them to form pores in the cell membrane. Additional caspase-1-mediated processing leads to the maturation of IL-1β and IL-18, which can exit the cell through the gasdermin pores. The caspase-mediated modes of cell death are of paramount importance for health and disease [23]. Accordingly, caspase-dependent cell death and innate immune reactions are potential targets for the development of new therapeutic approaches in many diseases [23]. Targeted therapies require in-depth knowledge of the particular caspases [24], which, despite extensive research over more than thirty years, is still incomplete for some of the caspases with presumable pathological relevance.

In this review, we focus on caspase-5 and discuss features that make it unique among caspases. Caspase-5 is upregulated at the mRNA level and activated at the protein level by lipopolysaccharide (LPS), a critical molecule in infections with Gram-negative bacteria. The *Caspase 5* (*CASP5*) gene is the product of a gene duplication in the evolutionary lineage leading to humans, with *CASP4* being its closest relative.

## 2. The Structure of Caspase-5 Gene and Proteins

Caspase-5 belongs to the subfamily of proinflammatory proteases comprising caspase-1, -4 and -5 in humans and caspase-1 and 11 in mice. Mouse caspase-11 is the ortholog of both caspase-4 and caspase-5 [25]. Caspase-5 is one of the least characterized members of the caspase family, which is due to some of its unique features, as discussed below. Originally, cDNAs corresponding to partial caspase-5 mRNAs were reported under the names “ICErel-III” (interleukin-1β-converting enzyme-related III) [26] and “TY” [27]. A decade later, we reported the first cloning of the complete caspase-5 coding sequence [28]. As the initial experimental studies used recombinant caspase-5 forms, which lacked the amino-terminal segment of the protein, the results of these studies must be interpreted with caution. 

**Figure 1:**
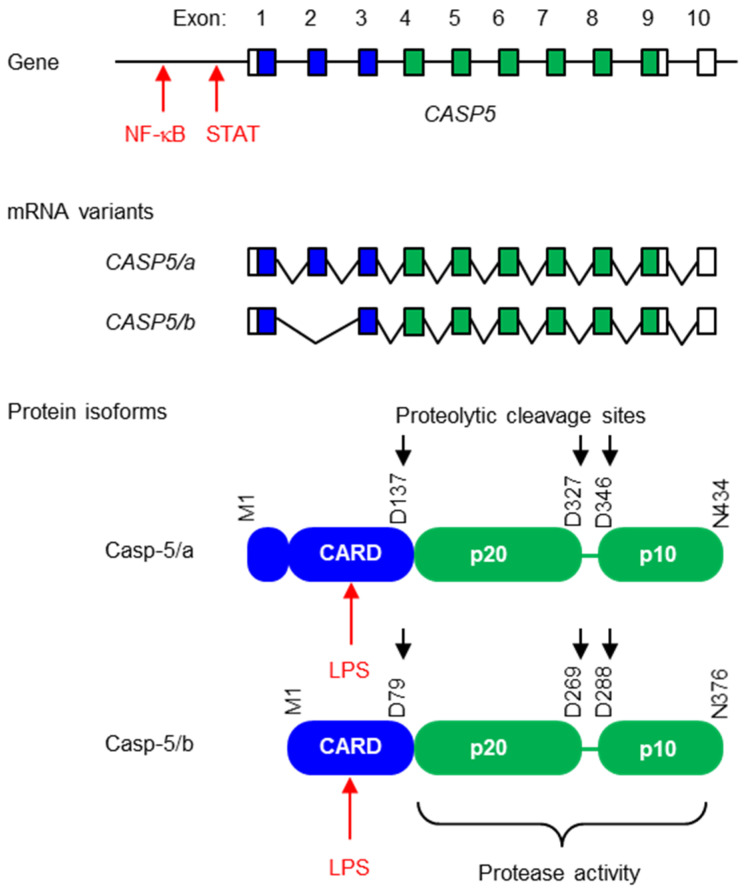
Structures of the human caspase-5 gene, mRNAs and proteins. The organization of exons of *CASP5* is schematically depicted with boxes representing exons. Segments colored blue and green encode the caspase recruitment domain (CARD) and the catalytic domain of the protein, respectively. White segments are non-coding regions at the 5’ and 3’ ends. The transcription factors NF-κB and STAT bind in the proximal promoter of *CASP5*. mRNAs undergo alternative splicing. The two main variants, *CASP5/a* and *CASP5/b*, are shown. The splice variants are translated to caspase-5 protein isoforms. Note that a short domain corresponding to a partial CARD is present at the amino-terminus of caspase-5/a. Black arrows point to aspartic acid residues (D) that are predicted to be sites of proteolytic cleavage during or after the activation of caspase-5 [14].

The human *Caspase 5* (*CASP5*) gene consists of 10 exons, with the start and stop codons being located in exons 1 and 9, respectively (Figure 1). Like in *CASP1* and *CASP4*, the last exon of *CASP5* lacks a coding sequence, but the distance between the stop codon and the last exon–exon junction is clearly below the threshold of 50 nucleotides [29], which is why breakdown of *CASP5* mRNA via nonsense-mediated decay is not expected. Alternative splicing of the pre-mRNA leads to at least six mRNA variants of human *CASP5,* termed *CASP5/a* through *f* [28]. The two main variants are *CASP5/a* and *CASP5/b* [28], but different cell types may have different preferences for CASP5 splicing. *CASP5/a* mRNA contains all the exons, while *CASP5/b* is structurally equivalent to the full-length mRNAs of *CASP1* and *CASP4*, as it lacks the second exon that is specifically present in *CASP5*. Transcriptome analyses of human tissues and organs showed the expression of *CASP5* in blood cells, spleen, lung, intestine and colon (https://www.gtexportal.org/home/gene/CASP5, last accessed on 28 March 2024).

Exon 1 of *CASP5* is a homologous exon 1 of mouse *Casp11* and human *CASP4* and *CASP1* [28]. The proximal promoter on the 5’-side of this exon displays significant sequence similarity to the mouse *Casp11* promoter, which was reported to be critical to the upregulation of caspase-11 in response to LPS and interferon–gamma [30]. The sequence motifs involved in binding of nuclear factor-κB (NF-κB) and signal transducer and activator of transcription (STAT) are conserved in the promoter of *CASP5* and exhibit a better fit to the consensus motifs than homologous sites in the promoter of *CASP4* [28]. 

**Figure 2:**
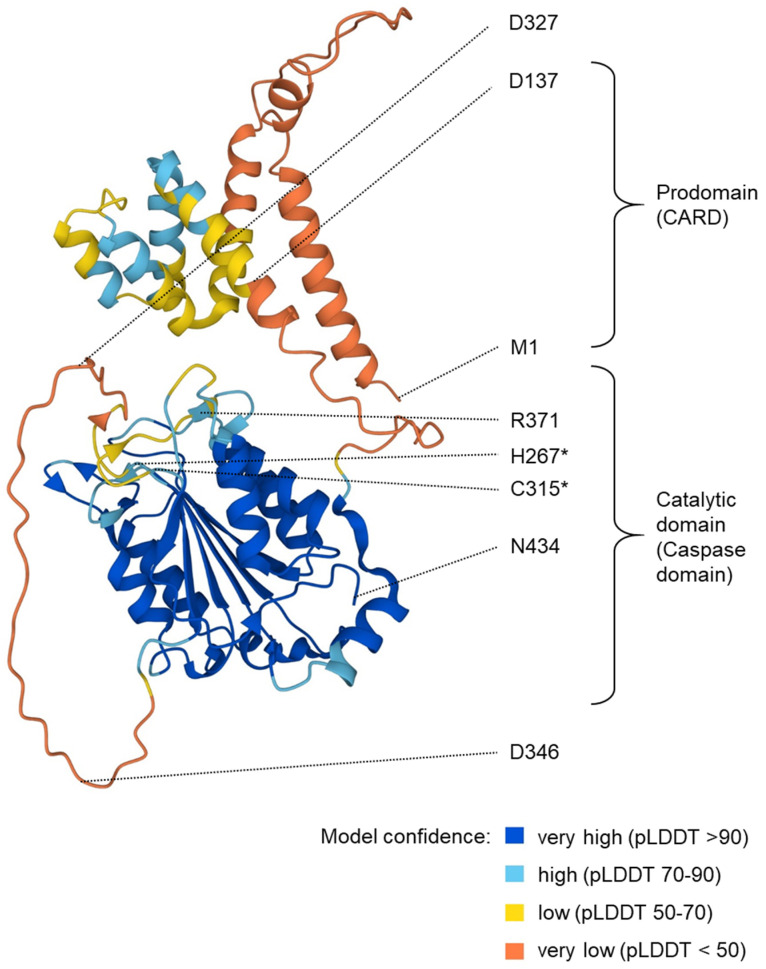
Structure prediction for human caspase-5/a. The image shows a structure prediction available at https://alphafold.ebi.ac.uk/entry/P51878 (last accessed on 3 April 2024). The positions of particular residues are indicated. Asterisks (*) mark the residues (H267 and C315) of the catalytic dyad. R371 is involved in substrate binding. Aspartate (D) residues at the predicted proteolytic cleavage sites are highlighted. pLDDT: predicted local distance difference test.

The caspase-5 protein isoforms are primarily the translation products of the various *CASP5* mRNA variants. Caspase-5/a, b (Figure 1) and f [28] consist of an amino-terminal CARD and the caspase domain (Figure 2). A shorter isoform lacking the CARD is encoded by the *CASP5/c* mRNA, whereas *CASP5/d* and *CASP5/e* mRNAs code for short and probably non-functional proteins devoid of defined domains. However, another isoform, previously denoted as capase-5-S, appears to be translated from an in-frame ATG downstream of the primordial start codon [28]. Overexpression studies in HEK293 cells suggest that the caspase-5/a and b isoforms and, to a lesser extent, capase-5-S, are able to induce cell death, including the activation of caspase-3. Fragments corresponding to the p10 subunit were detected, indicating that proteolytic cleavage occurs at one or both processing sites between p20 and p10 (Figure 1 and Figure 2) [28].

## 3. The Function of Caspase-5 in the Response to Gram-Negative Bacteria

### 3.1. LPS Regulates the Expression of Caspase-5

**Figure 3:**
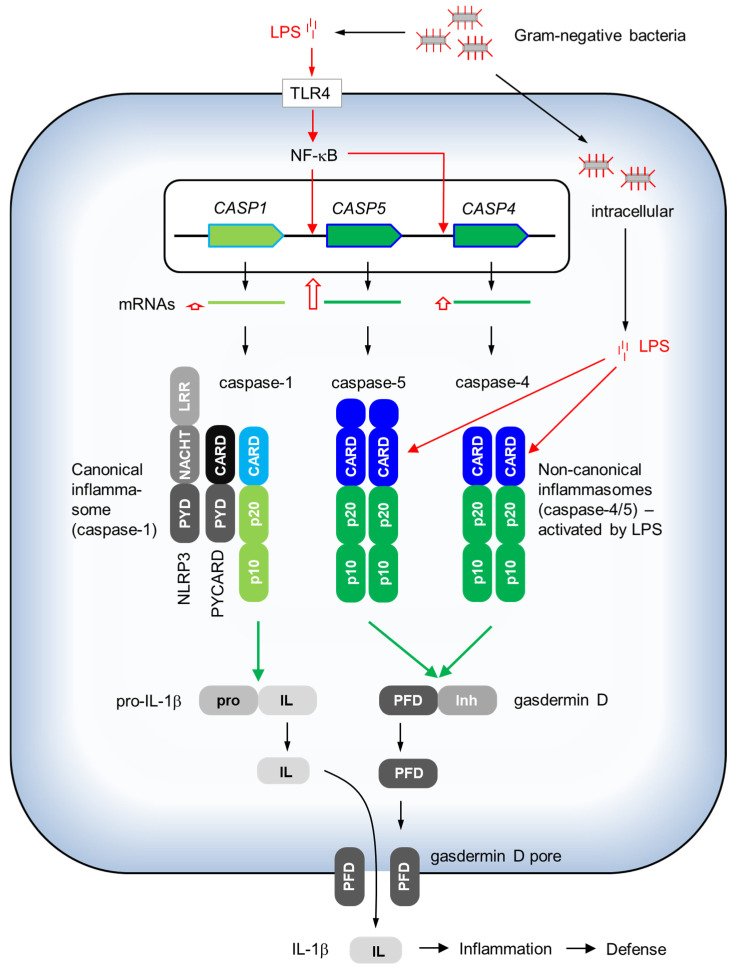
Regulation and function of caspase-1, -4 and -5 in response to infection with gram-negative bacteria. The genes *CASP1*, *CASP5* and *CASP4* are arranged in tandem. The transcription of *CASP5* and, to a lesser degree, *CASP4* is induced by LPS-induced NF-κB signaling. Caspase-1 is activated by canonical inflammasomes such as the one including NLRP3, whereas caspase-4 and caspase-5 form non-canonical inflammasomes upon the binding of intracellular LPS to their CARD. The preferred substrate of caspase-1 is pro-interleukin-1β. Caspase-4 and caspase-5 cleave the inhibitory (Inh) domain off the pore-forming domain (PFD) of gasdermin D, facilitating the formation of a pore through which mature IL-1β is secreted to activate inflammation and anti-bacterial defense. The domains of caspases are colored, as defined in Figure 1. TLR4: toll-like receptor 4.

The primary function of caspase-5 is to induce inflammation upon infection with intracellular Gram-negative bacteria (Figure 3). Lipopolysaccharide originating from bacteria is detected by toll-like receptor 4 (TLR4) at the cell membrane, leading to the activation of NF-κB signaling. As mentioned above, *CASP5* contains an NF-κB binding site in its proximal promoter. The transcription of *CASP5* is massively upregulated upon exposure to LPS both in cultured cells [31,32,33] and in vivo [28]. Healthy human volunteers who intravenously received a low dose of LPS showed peak expression levels of more than 10-fold compared to basal levels, whereas *CASP4* and *CASP1* were only weakly upregulated in the range of 1.5- to 2.5-fold in peripheral blood mononuclear cells (PBMCs) [28]. The increase in *CASP5* expression is delayed relative to that of *IL1B* both in the monocytic cell line THP-1 [31] and in PBMCs in vivo [28].

Importantly, the elevation of *CASP5* mRNA upon exposure to LPS is replicated by the LPS induction of *Casp11* mRNA in mice, suggesting that, at least in this regard, the mouse is a better model for human *CASP5* than for *CASP4*. As most of the research studies on the regulation and functions of proinflammatory caspases use mice, it is also important to note that the mouse strain 129 carries a mutation that inactivates the *Casp11* gene [34]. *Casp1* knockout mice that were generated by targeting embryonic stem cells of the strain 129 also carry the mutation of *Casp11*. The fact that the presumed *Casp1* knockouts were actually *Casp1*/*Casp11* knockouts was reported in the year 2011, leading to the re-interpretation of many previous knockout mouse studies [34].

### 3.2. LPS Activates Caspase-5 in the Non-Canonical Inflammasome

The cellular response to LPS does not only include the induction of gene expression but also the initiation of reactions at the protein level [35]. When Gram-negative bacteria invade the cytosol, LPS can be directly detected by murine caspase-11 and human caspase-4 and -5, but not by caspase-1 [36,37]. In line with this role of caspase-11 as a pattern recognition receptor for LPS, caspase-11, but not caspase-1, is essential for LPS-induced lethality in a mouse model [34]. The roles of caspase-11/4/5 as pattern recognition receptors deviates from the roles of other caspases, which are either activated by the homotypic interactions of their prodomains with other proteins or by proteolytic activation, as discussed above (Section 2). In an elegant study, Shi et al. demonstrated that LPS, and particularly its lipid A moiety, bind to the CARD of caspase-11/4/5, leading to the oligomerization and activation of the respective caspase [36]. This study was performed with hexa-acylated lipid A, and the failure of underacylated lipid A from *Rhodobacter sphaeroides* to activate caspase-11 suggested a critical dependence on the lipid A acylation [36]. However, another study demonstrated that human caspase-4 is activated by both hexa-acylated and tetra-acylated lipid A, which indicated a difference in the LPS response of humans and mice. The responsiveness of human caspase-5 could not be determined in the study of Lagrange et al. [32] and remains an important open research question, with potential implications for the translation of findings in mouse models to human diseases. Later studies showed that the roles of human caspases-4/-5 and mouse caspase-11 as sensors for LPS depend on guanylate-binding proteins (GBPs), which assemble on the surface of cytosolic Gram-negative bacteria and serve as polyvalent platforms required for caspase activation [38,39,40].

The binding of LPS to caspase-11/4/5 induces the formation of a non-canonical inflammasome, which, as opposed to canonical inflammasomes, contains neither the adaptor protein ASC/PYCARD nor a pattern recognition receptor that is different from a caspase [35,41]. The efficient assembly of the non-canonical inflammasome in response to cytosolic LPS depends on the auto-proteolytic cleavage between the p20 and p10 subunit, with the latter remaining in the complex [42]. This autoprocessing is also required for full activation and for the cleavage of the preferred substrate of caspase-11/4/5, namely gasdermin D (GSDMD) [41,43,44,45,46]. GSDMD belongs to an evolutionarily conserved family of pore-forming proteins, known as gasdermins. GSDMD is expressed as cytosolic protein composed of an amino-terminal pore-forming domain, which is inhibited by a carboxy-terminal domain of the same protein. Caspase-11, -4 and -5 cleave between the two GSDMD domains and thereby release the amino-terminal domain. The latter forms pores in the cell membrane to allow for the release of proteins including, most importantly, a product of canonical inflammasomes, mature interleukin 1-beta.

Canonical inflammasomes consist of caspase-1, ASC/PYCARD and a sensor/pattern recognition protein, such as NLRP1, NLRP3, NLRP6, NLRC4/NAIP, AIM2, CARD8, or pyrin [41]. The inflammasome-associated sensor proteins respond to aberrant proteolytic activities, ribotoxic stress, bacterial lipoteichoic acid, flagellin or components of bacterial type III secretion systems, viral double-stranded RNA and DNA and other stimuli [41]. In the context of the functions of caspase-5, the NLRP3 inflammasome is considered to be primarily important for the activation of caspase-1 and the subsequent processing of pro-IL-1β. The resulting mature interleukin-1β is secreted through caspase-5-generated GSDMD pores to activate inflammation and anti-bacterial defense (Figure 3). Interestingly, the paper in which the term “inflammasome” was first used proposed that caspase-5 forms a complex with caspase-1, ASC/PYCARD and NALP1/NLRP1 [47], but the involvement of caspase-5 in this inflammasome was not confirmed [48]. 

### 3.3. Mechanisms of Sensing Cytosolic LPS and Oxidized Phospholipids

Recently, NLRP11 was reported to act as a pattern recognition receptor for cytosolic LPS in macrophages [49]. NLRP11 binds LPS and caspase-4 to form an inflammasome in HEK293T cells. Among the caspase-5 isoforms, only caspase-5/a was tested, and this isoform did not interact with NLRP11 [49]. Of note, the mouse does not have an NLRP11 ortholog, and human NLRP11 is predominantly expressed in the testis [50]. Another recent report showed that the murine homolog of caspase-5, i.e., caspase-11, can interact with NLRP3. This interaction depends on the binding of LPS to caspase-11 and on the detection of bacterial RNA by NLRP3, together leading to the assembly and activation of an NLRP3 inflammasome in macrophages [51]. Furthermore, the receptor protein Nur77 binds cytoplasmic LPS and activates a non-canonical NLRP3 inflammasome [52]. Recently, adipose triglyceride lipase (ATGL), also known as patatin-like phospholipase domain containing 2 (PNPLA2) [53], was reported to suppress the noncanonical inflammasome by hydrolyzing LPS [54]. Thus, the model of the caspase-5-mediated response to Gram-negative bacteria (Figure 3) shows only the central processes, while additional levels of regulation or interdependencies with other pathways relevant for specific cell types are not covered.

Importantly, caspase-11 and caspase-4 bind not only LPS but also oxidized 1-palmitoyl-2-arachidonoyl-sn-glycero-3-phosphorylcholine (oxPAPC) [55,56]. The oxidized form of the endogenous phospholipid is known as a modulator of inflammation [57]. Apparently contradictory results have been reported, suggesting either the proinflammatory [55] or anti-inflammatory [56] effects of oxPAPC binding to caspase-11. An interaction between oxPAPC and caspase-5 has been proposed but remains to the investigated [58]. As oxPAPC and potentially other endogenous ligands may modify the activity of pro-inflammatory caspases, further studies of these interactions are warranted. 

As discussed above, the majority of studies utilize caspase-11 in the mouse model and draw conclusions regarding human cells and tissues, mainly testing caspase-4. Caspase-5 was confirmed to bind LPS and to cleave GSDMD [36,44], but its importance in vivo and in isolated cells is not fully understood. In some experimental systems, researchers concluded that caspase-4 is more important than caspase-5 for anti-bacterial defense mediated by non-canonical inflammasomes [59]. In another study, caspase-5 was shown to respond to intracellular LPS when cells were infected with live bacterial, but not when isolated LPS was transfected into cells [60]. 

### 3.4. Substrates of Caspase-5

Caspase-5 cleaves and activates GSDMD, but it can also target other proteins. Pro-IL-18 can be cleaved by caspase-5 with comparably low efficiency [13]. Another study showed that caspase-5 cleaves pro-IL-1β after residue D27 instead of D116, which is targeted by caspase-1, leading to deactivation rather than the activation of IL-1 signaling [14]. Recently, pro-IL-1α was reported to be cleaved by human caspase-5, but not by caspase-1 and caspese-4 [61]. The expression of *CASP5* increased in senescent cells and played an essential role in establishing the IL-1α-dependent senescent-associated secretory phenotype (SASP) [61]. 

The overexpression of caspase-5 in HEK293 cells results in the proteolytic activation of caspase-3 [28], and caspase-4 cleaves caspase-7 [62], suggesting links between caspase-5 and caspase-3/7-mediated apoptosis, if the preferred cleavage of GSDMD does not suffice to induce pyroptosis. However, the physiological significance of these cleavages is not known [14]. Likewise, caspase-5-mediated cleavage of the transcription factor Max after glutamic residues [63] has not been confirmed in vivo.

## 4. The Evolution of Caspase-5 and Related Caspases

**Figure 4:**
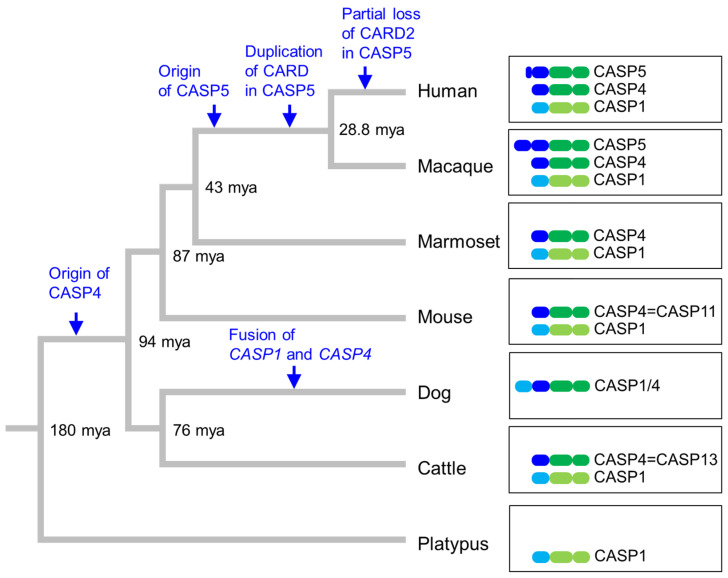
Evolution of caspase-1, -4 and -5 in mammals. Divergence times of lineages (mya, million years ago) are indicated [67]. *CASP1/4* denotes the protein encoded by the gene that evolved through the fusion of *CASP1* and *CASP4* genes [3,68]. *CASP11* [64] and *CASP13* [65] are the orthologs of *CASP4* in mouse and cattle, respectively. The domain organization of caspases is shown on the right with domains being colored as defined in Figure 1 and Figure 3. Species: Human (*Homo sapiens*), macaque (*Macaca fascicularis*), marmoset (*Callithrix jacchus*), mouse (*Mus musculus*), dog (*Canis lupus*), cattle (*Bos taurus*) and platypus (*Ornithorhynchus anatinus*).

The presence of two orthologs, caspase-4 and caspese-5, in humans, as opposed to the presence of only caspase-11 in the main biomedical model species, the mouse, represents a challenge for research and points to a role of caspase diversification in the evolutionary lineage leading to humans. The evolution of pro-inflammatory caspases can be inferred from the distribution of the corresponding genes in phylogenetically diverse species and their known relationships [3]. Based on the data available in GenBank, the presence of relevant caspase genes is depicted at the end nodes of a phylogenetic tree of mammals (Figure 4). Humans and macaques and other species of the clade *Catarrhini* (old world anthropoids/monkeys) have *CASP1*, *CASP4* and *CASP5* genes, which are located in tandem (on chromosome 11q22.3 of the human genome) (Figure 4). By contrast, the next outgroup (*Platyrrhini*, new world monkeys), represented by the marmoset, has only *CASP1* and *CASP4*. This difference suggests that *CASP5* originated in stem *Catarrhini*. Orthologs of *CASP1* and *CASP4* are also present in the mouse and cattle. For reasons related to the history of research, the single ortholog of *CASP4/5* is called *CASP11* in the mouse [64] and *CASP13* in the cattle [65]. In the dog and other carnivorans *CASP1* and *CASP4* genes are fused into a single gene in which the promoter and exons 1 and 2 of *CASP1* are followed by exons 2 through 9 of *CASP4* [3]. Alternatively spliced mRNA variants encode a protein with two CARDs and another protein with only one CARD [3]. This evolutionary gene recombination inspired an interesting protein design study in which the LPS receptor function of human caspase-4 was combined with the pro-interleukin-1β processing activity of caspase-1 [66]. The platypus, representing the basal mammalian clade of monotremes, and non-mammalian amniotes (reptiles and birds) have only one ortholog of human *CASP1*, *CASP4* and *CASP5*, termed *CASP1* (Figure 4). Taken together, this species distribution of caspases suggests that a single ancestral gene underwent two duplications to give rise to *CASP1*, *CASP4* and *CASP5* in humans and closely related primates.

Whether the direct binding of caspases to LPS evolved in mammals or earlier in evolution is currently not known. The ability of a zebrafish caspase, termed caspy2, to bind LPS through its prodomain [69,70] points to an early origin of caspase–LPS interactions. Of note, several mammals, but not humans and mice, have an additional evolutionary ancient caspase, denoted as caspase-15, which is related to caspase-1, -4 and -5 [68]. The mechanism of activation and the function of caspase-15 are not known. Furthermore, caspase-12 is phylogenetically closely related to caspase-1, -4 and -5 and undergoes processing upon the LPS treatment of mouse cells [71]. However, the physiological significance of caspase-12 is uncertain and the human caspase-12 gene has undergone pseudogenization [72,73].

*CASP5* differs from *CASP1* and *CASP4* by the presence of a unique exon between exon 1 and the CARD-encoding exon 3 (Figure 1). A homolog of this exon 2 is also present in other catarrhine primates. Interestingly, exon 2 of macaque *CASP5* encodes a complete CARD (https://www.ncbi.nlm.nih.gov/gene/102120532, last accessed on 28 March 2024) so that macaque *CASP5* is predicted to encode a caspase with two CARDs (Figure 4). In the lineage leading to humans, exon 2 was truncated, so that the *CASP5/a* mRNAs contains a partial CARD in addition to the complete CARD. Whether the deviation from the canonical structure of caspase-4 endows caspase-5/a with special properties remains to be investigated.

## 5. Roles of Caspase-5 in Human Diseases

As LPS induces the expression of *CASP5* and activates caspase-5 within the non-canonical inflammasome, the primary function of caspase-5 is the initiation or amplification of the cellular response to LPS [74,75]. Infections with Gram-negative bacteria, such as *Escherichia coli* and *Vibrio cholerae*, can cause lethal diseases [34], and the appropriate initiation of an antibacterial defense counteracts disease both in mouse models and humans. Conversely, excessive inflammation is a major contributor to sepsis and inflammatory bowel disease (IBD). The mouse ortholog of capase-5, caspase-11, is critical for GSDMD activation, the release of IL-1β and IL-18 and tissue damage in a mouse model of endotoxemia/sepsis [76]. Caspase-11, expressed in macrophages/monocytes, as compared to dendritic cells, neutrophils and intestinal epithelial cells, plays the dominant role in causing pathological manifestations of LPS shock [76]. In contrast, both monocytes/macrophages and neutrophils contribute to caspase-11-dependent host defense against the intracellular bacterium, *Burkholderia thailandensis* [76]. The expression of human caspase-4 in transgenic mice caused elevated sensitivity to LPS challenge [77].

Gram-negative bacteria from the intestine can enter the tissue if the epithelial barrier is disturbed in IBD. Subsequently, cell-invasive bacteria activate caspase-4 and -5 [75,78,79], which are implicated in the development of chronic, relapsing inflammation. The growth of *Legionella pneumophila*, the causative agent of Legionnaires’ pneumonia, in human macrophages was restricted by the ectopic expression of caspase-5. The mechanism of this restriction is not completely known but was suggested to involve the dephosphorylation of cofilin to modulate the polymerization of actin and subsequently the fusion of the *L. pneumophila* vacuole with lysosomes [80]. According to a recent paper, caspase-5 is directly involved in the dysfunction of the intestinal epithelial barrier in response to LPS [81]. Using immortalized Caco-2 cells, which are derived from human colorectal adenocarcinoma as a model, caspase-5 was reported to contribute, along with sorting nexin 10 (SNX10) and the PIKFYVE kinase, to the release of LPS from internalized bacterial outer membrane vesicles into the cytosol. Subsequent signaling, also involving caspase-5, leads to the downregulation of E-cadherin and impairment of the barrier function [81,82]. A role of caspase-5 in the response to membrane vesicles from *Pseudomonas aeruginosa* vesicles was also reported for monocytes [83].

Unexpectedly, the non-canonical inflammasome has been implicated in the response to bacteria without LPS, such as *Ehrlichia chaffeensis* [84], and in non-infectious diseases. Caspase-5 mRNA is upregulated 20-fold in lesional skin as compared to nonlesional skin in patients with psoriasis [85]. The bacterial load on psoriatic skin is generally low due to the efficient activation of anti-microbial defense. *CASP5* mRNA could be induced by LPS in an NF-κB-dependent manner in PBMCs but not in keratinocytes [85]. Another paper reported on the expression and activity of caspase-5 in psoriasis [86]. However, due to the potential cross-reactivity of antibodies, immunodetection studies of caspase-5 must be interpreted with caution. The mouse ortholog of caspase-5, caspase-11, is implicated in the allergic airway inflammation [87]. Recently, caspase-4 and -5 were reported to be activated by heme, possibly contributing to inflammation in bacterial sepsis, malaria and sickle cell disease [88]. The mechanism of activation and the interaction of caspase-5 with caspase-1, which is also activated by heme [89], remain to be determined.

The human *CASP5* gene carries several single nucleotide polymorphisms (SNPs). In contrast to SNPs in *CASP12* [90], which does not encode a functional protease in humans [73], SNPs in *CASP5* have not yet been linked to differential responsiveness to LPS. However, one SNP was reported to be associated with rheumatoid arthritis [91], and mutations of *CASP5* were detected in cancers [92,93]. The rs3181320*C allele of *CASP5* (exon 2), leading to an amino acid substitution, was suggested as a potential risk factor for cancer [94]. A stretch of 10 adenines at the beginning of exon 3 (encoding the CARD) was reported to carry frame-shift mutations in the form of one-nucleotide deletions (A9) or insertions (A11) in tumors of the stomach and colon [92]. This site corresponds to human SNP rs112680102. According to the currently available data (https://www.ncbi.nlm.nih.gov/snp/rs112680102, last accessed on 28 March 2024), the *CASP5* A11 allele occurs at frequencies of 4.88% and 2.75% (ALFA allele frequencies according to release version: 20230706150541) in Europeans and Africans, respectively. The clinical significance of the variant alleles of *CASP5* is unknown. Recently, homozygosity for the c.1300C-T transition (c.1300C-T, NM_001136112.3), resulting in a missense mutation (R434X), was reported to be linked to a Werner syndrome-like progeroid disorder [95], but the contribution to this disease has not been confirmed yet (https://omim.org/entry/602665, last accessed on 28 March 2024). Taken together, several studies in model systems and clinical data point to the role of caspase-5 in a larger range of diseases. However, these putative roles must be critically assessed in further studies.

Caspase-5-dependent processes are potential targets for clinical therapies. The intracellular sensing of LPS by the non-canonical inflammasome has been discussed as a target in the therapy of sepsis [96]. Small chemical inhibitors of caspases have been tested in preclinical and clinical studies, but none of the candidate drugs has been successful so far [97,98]. Therefore, it may be more promising to target processes downstream of caspase activity, such as the formation of pores by GSDMD [99,100,101] and the signaling via IL-1 cytokines [102] or other pro-inflammatory proteins released through GSDMD pores [103]. To the best of our knowledge, therapies targeting specifically caspase-5 have not been tested yet.

## 6. Conclusions

Caspase-5 is a unique human caspase that is both transcriptionally upregulated and post-translationally activated by LPS. The ortholog of caspase-5 in the mouse is caspase-11. However, due to the presence of caspase-4, a structurally similar paralog to caspase-5 in humans, it is difficult to translate findings from mouse studies to human conditions. Accordingly, the contribution of caspase-5 to the defense against Gram-negative bacteria and to the response to LPS in sepsis are only incompletely understood at present. A better characterization of the functions of caspase-5 likely depends on the improvement of in vitro models, the careful design of experiments, taking into account the time course of LPS-induced *CASP5* expression, and clinical studies in which genetic polymorphisms of *CASP5* are considered.
